# Time-dependent growth of crystalline Au^0^-nanoparticles in cyanobacteria as self-reproducing bioreactors: 2. *Anabaena cylindrica*

**DOI:** 10.3762/bjnano.7.30

**Published:** 2016-03-02

**Authors:** Liz M Rösken, Felix Cappel, Susanne Körsten, Christian B Fischer, Andreas Schönleber, Sander van Smaalen, Stefan Geimer, Christian Beresko, Georg Ankerhold, Stefan Wehner

**Affiliations:** 1 Universität Koblenz-Landau, Institut für Integrierte Naturwissenschaften, Abteilung Physik, 56070 Koblenz, Germany; 2 Universität Bayreuth, Lehrstuhl für Kristallographie, 95440 Bayreuth, Germany; 3 Universität Bayreuth, Zellbiologie / Elektronenmikroskopie, 95440 Bayreuth, Germany; 4 Hochschule Koblenz, RheinAhrCampus Remagen, Optics and Laser Engineering, 53424 Remagen, Germany

**Keywords:** biosynthesis, gold nanoparticles, laser-induced breakdown spectroscopy (LIBS), transmission electron microscopy (TEM), X-ray powder diffraction (XRD)

## Abstract

Microbial biosynthesis of metal nanoparticles as needed in catalysis has shown its theoretical ability as an extremely environmentally friendly production method in the last few years, even though the separation of the nanoparticles is challenging. Biosynthesis, summing up biosorption and bioreduction of diluted metal ions to zero valent metals, is especially ecofriendly, when the bioreactor itself is harmless and needs no further harmful reagents. The cyanobacterium *Anabaena cylindrica* (SAG 1403.2) is able to form crystalline Au^0^-nanoparticles from Au^3+^ ions and does not release toxic anatoxin-a. X-ray powder diffraction (XRD), transmission electron microscopy (TEM) and laser-induced breakdown spectroscopy (LIBS) are applied to monitor the time-dependent development of gold nanoparticles for up to 40 hours. Some vegetative cells (VC) are filled with nanoparticles within minutes, while the extracellular polymeric substances (EPS) of vegetative cells and the heterocyst polysaccharide layer (HEP) are the regions, where the first nanoparticles are detected on most other cells. The uptake of gold starts immediately after incubation and within four hours the average size remains constant around 10 nm. Analyzing the TEM images with an image processing program reveals a wide distribution for the diameter of the nanoparticles at all times and in all regions of the cyanobacteria. Finally, the nanoparticle concentration in vegetative cells of *Anabaena cylindrica* is about 50% higher than in heterocysts (HC). These nanoparticles are found to be located along the thylakoid membranes.

## Introduction

Precious metallic nanoparticles are of steadily increasing interest since there are widespread possibilities of usage [1–3]. Especially Au^0^-nanoparticles are used, e.g., in heterogeneous catalysis [4–6] or in various medical applications [7–8].

There is a growing field of research in microbiological approaches for the production of nanoparticles since more than 30 years [9]. Instead of using conventional chemical pathways the implementation of microbiological methods is environmentally friendly, especially when the bioreactor itself is harmless and in general since no further harmful reagents are needed, even sustainable [10–12]. Therefore biological pathways utilize for example plants [13–14], bacteria [15–20] and fungi [21–22].

In living organisms the biosynthesis of nanoparticles is often done by enzymatic processes which convert in the end a dissolved ion into a structurally stable state. Later they may be excreted from the cell [23]. For further use the nanoparticles have to be separated from all the unavoidable molecules present in living organisms, like the biological capping and reducing ligands, e.g., enzymes, chlorophylls, peptides or polyphenols [10,24]. This task is typically quite challenging. Reduction of the toxic metal ions with or without subsequently excretion of the metal atoms is one possibility for a living organism to handle harmful environments [25–26].

In addition reduction of metal ions may also occur by other macromolecular cell components like proteins or sugars. These processes mainly occur outside the cells in the exopolysaccharides, also named extracellular polymeric substance or extracellular polysaccharides. As they all are commonly abbreviated EPS, this phrase is used throughout this paper to specify this region [23,27]. Cyanobacteria’s heterocysts (HC) are surrounded by a similar coating, the heterocyst polysaccharide layer (HEP) [28]. In vivo formation of nanoparticles in these layers was, e.g., reported for *Anabaena* sp. in an earlier study in the setup used here [29]. Bioreduction can also occur in vitro within cell extracts [19–20,30].

It is well known that some microbiological metabolisms are able to reduce dissolved metal ions to their native state, e.g., the bioreduction from Pt^4+^ to platinum in the native state [19–20]. The reduction of Au^3+^ by means of enzymes like hydrogenase [31–33] and nitrogenase [23] is another prominent example. In case of nitrogenase the reduction of elemental nitrogen to ammonium [34–35] generates the electrons which are required for the reduction of harmful (possibly cytotoxic) metal ions to their zero valent form.

As nitrogenase is a very oxygen-sensitive enzyme it has to be protected from oxygen [36]. Some cyanobacteria form therefore so-called heterocysts [37–38]. Compared to the vegetative cells (VC) heterocysts are relatively large cells. The formation of heterocysts is upregulated by media, which contain too little bound nitrogen like nitrates [37–38].

Cyanobacteria offer several advantages as a promising nanoparticle-producing agent. Firstly, they are ubiquitously present and therefore able to survive in nearly all natural habitats. Moreover, an unintended liberation would not endanger the environment. Furthermore cyanobacteria are undemanding organisms, they are easy to handle in terms of cultivation as well as bioreduction. Whereas most of other organisms require anaerobic conditions and additional reagents like electron donors [13–16,18,21,39], cyanobacteria are capable to reduce metal ions in aerobic conditions and without any further reducing agent within a short time [23,28,30,40–42]. In spite of all these advantages still little is known about non-hazardous cyanobacteria as self-reproducing bioreactors for the production of nanoparticles.

Brayner et al. showed that gold nanoparticles are enriched around heterocysts for *Anabaena flos-aquae* strain ALCP B24 from the culture collection of MNHN (Muséum National d'Histoire Naturelle) [23]. Here we have used the strain *Anabaena cylindrica* 1403.2 (Algensammlung Göttingen, SAG), which contains more heterocysts than a comparable *Anabaena flos-aquae* strain (SAG 30.87). When heterocysts are important for the biosynthesis of nanoparticles, as written in literature, more heterocysts per organism should be equal to an enhanced nanoparticle production. However our studies on *Anabaena* sp. (SAG 12.82) concerning bioreduction of Au^3+^ have shown that for this species and given environmental conditions the most gold nanoparticles can be finally found in vegetative cells and only a minor part in heterocysts [29]. Only in the beginning (first minutes after incubation with Au^3+^ ions started) the HEP of heterocysts is the preferred region for the formation of nanoparticles, while at the same time no nanoparticles were recorded inside vegetative cells.

Here we demonstrate the ability of *Anabaena cylindrica* 1403.2 to reduce Au^3+^ ions into their native state. The subsequent growth of crystalline gold nanoparticles has been investigated. In accordance to the study with cyanobacteria *Anabaena* sp. (SAG 12.82) [29] we show in this study for another cyanobacterium, that the vegetative cells are more important as location for nanoparticle biosynthesis than the heterocysts.

In this study we have undertaken first steps to quantify the uptake of gold into the cyanobacteria in various ways: Laser-induced breakdown spectroscopy (LIBS) shows that the higher the concentration of gold ions in the culture the larger is the amount of gold found in the biomass. From the average size of nanoparticles determined by X-ray powder diffraction (XRD) and the number of nanoparticles recorded by transmission electron microscopy (TEM) the average uptake of the cyanobacteria can be calculated. Using image processing software the size and their distribution of formed nanoparticles can be read out directly from TEM images. This was done for the early and later stages of the incubation process at the characteristic regions of the cyanobacteria.

## Materials and Methods

### Cultures

The cultures used throughout this study were grown in 250 mL Erlenmeyer flasks containing 150 mL modified Bold's Basal Medium (BBM, pH 6.8) with 50% less nitrate and without vitamins [43–45]. The cultures were shaken continuously horizontally (Labworld Orbital Shaker 20) and placed in a temperature-controlled incubator at 22 °C and illuminated with daylight spectrum (color of 4,200 K) in a 12 h-day-night-rhythm. The pH-value of cultures of *Anabaena cylindrica* is 7.3.

Four days before incubation the culture has been split in two. One part was incubated with an overall concentration of 0.8 mM Au^3+^ (HAuCl_4_·xH_2_O p. a., Carl Roth, Karlsruhe, Germany) dissolved in modified BBM, the other was cultivated untreated as reference.

For each intended measurement an aliquot volume of 2 mL was taken. Depending on the type of analysis, X-ray powder diffraction (XRD), transmission electron microscopy (TEM) or laser-induced breakdown spectroscopy (LIBS), it was processed differently; see Supporting Information File 1 for more details. As the first centrifugation step takes 15 minutes, we assign for all data points a temporal uncertainty of 10 minutes. Biosynthesis will stop at any time between taking the sample from the flask and separating the biomass from the supernatant after centrifugation. Samples for XRD, TEM and LIBS are based on the biomass separated from the aliquot; see Supporting Information File 1 for a detailed description of these processes.

### X-ray powder diffraction

For XRD the washed biomass was placed on a homemade sample holder, whose main component is a 1 mm thick disk of Si(977) oriented single crystal, 25 mm in diameter. As XRD instrument a Philips X’pert was used to show the crystallinity of the formed nanoparticles and to determine their average size.

### Transmission electron microscopy

For TEM analysis samples were chemically fixed using glutaraldehyde and osmium tetroxide, dehydrated and embedded in epoxy resin according to standard procedures, see Supporting Information File 1 for a detailed description of the method. Ultrathin sections (about 60 nm thick) were analyzed in a Zeiss EM 902A transmission electron microscope. These images reveal the spatial distribution of the nanoparticles inside the cells as it was in the moment when their biosynthesis was stopped.

### Laser-induced breakdown spectroscopy

In recent years UV–vis spectroscopy has often been used for the in situ observation of nanoparticle or nanocluster development [46–48] of various metals, e.g., palladium, gold and silver [46]. Although UV–vis spectroscopy based methods have been developed to determine the size of nanoparticles resp. nanoclusters in solution [47–48]. UV–vis spectroscopy was also tested for the type of system used here in an early stage, but since superposition of the signals from components in the modified BBM or the cyanobacteria themselves together with gold have been found, no useful data for the depletion of gold ions from the solution could be collected in this case [29]. Therefore another spectroscopic element sensitive method – laser-induced breakdown spectroscopy (LIBS) – was established for such kind of suspensions.

The basic idea of LIBS is to ionize a limited part of the sample to a small plasma of less than one millimeter size by an intense short laser pulse of some nanoseconds and to analyze spectroscopically the emitted element specific plasma light by a fast array spectrometer during the decay of the plasma. The wavelength and the decline of intensity of characteristic emission peaks allow qualitative and sometimes quantitative analysis of the present elements. The method of LIBS is described in more detail elsewhere [49–54]. For LIBS measurements, the separated biomass was dried and placed without any further processing in the apparatus. A LIBS micro-plasma was produced with a homemade setup from commercially available components, see Supporting Information File 2 for a scheme of the setup. The LIBS setup arrangement consisted of a pulsed laser source, focusing optics, and Czerny–Turner spectrometers. As laser source a low-power passively Q-switched Nd:YAG laser (CryLas, model DSS1064-3000) at a wavelength of 1064 nm, a pulse energy of 2.5 mJ, a pulse duration of 2 ns (FWHM) and a repetition rate of 80 pulses per second was used. The plasma was generated by focusing the laser pulses on the sample surface by a quartz glass lens with a focal length of 20 mm. An irradiance of 2 GW per cm^2^ has been achieved.

The plasma emission was imaged by a combination of two quartz lenses (focal length 20 and 25 mm) into a glass fiber (core diameter 900 μm) guiding the light to a Czerny–Turner spectrometer (Ocean Optics, models MAYA 2000 Pro and USB2000). The MAYA 2000 Pro spectrometer covers a wavelength range from 190 to 420 nm with a spectral resolution of 0.1 nm, the USB2000 spectrometer a wavelength range from 190 to 860 nm with a spectral resolution of 0.3 nm. The CCD detectors were triggered by the laser pulses. The laser pulse energies were measured with a commercially available laser energy meter (Coherent, model LabMax-TOP).

The emitted light of the laser-induced plasma on the sample’s surface was analyzed by the spectrometers and processed by a self-programmed software tool on base of the LabVIEW software package (National Instruments). The data shown here are signals summed up over 100 individual measurements. Qualitative elemental analysis is performed by tracking element specific characteristic peaks as listed in databases [55]. A quantitative elemental analysis using LIBS spectra is only known for bulk materials and liquids for trace element analysis [52,56–57], but up to now unknown for diluted nanoparticles in a matrix. The herein presented semi quantitative analysis was performed by integrating the spectral peaks of interest after normalization to a background peak at 250.9 nm, originating from oxygen and nitrogen in ambient air [55]. The signal can therefore not be converted to the absolute mass of gold in the biomass, but larger peaks indicate more gold taken up by the cells, see below, for example.

### Image processing

TEM images show the spatial distribution of the formed nanoparticles within the cells. Since the magnification for each image is known the number of pixels could be covered easily in areas of nm^2^. The challenging problem is how many pixels represent a recorded nanoparticle. Since the grey scale is not changing stepwise (electron dense background) and electron microscopy images are always noisy, this could not be done precisely by eye and an image processing tool had to be used. A home-made software was developed and successfully applied to determine the area of each individual nanoparticle and to calculate, under the assumption of spherical nanoparticles, its diameter and volume. Since the relative error is decreasing with an increasing absolute number of pixels assigned to a nanoparticle in an image, significant values for the nanoparticles’ dimensions could be extracted only from TEM images of large enough magnification. In this study only TEM images of magnification larger than 50k could be used for this analysis. This limitation is the result of a combination of size of the produced nanoparticles, contrast differences within the image and resolution of the digital camera in the TEM setup used.

## Results

### Optical microscopy

**Figure 1:**
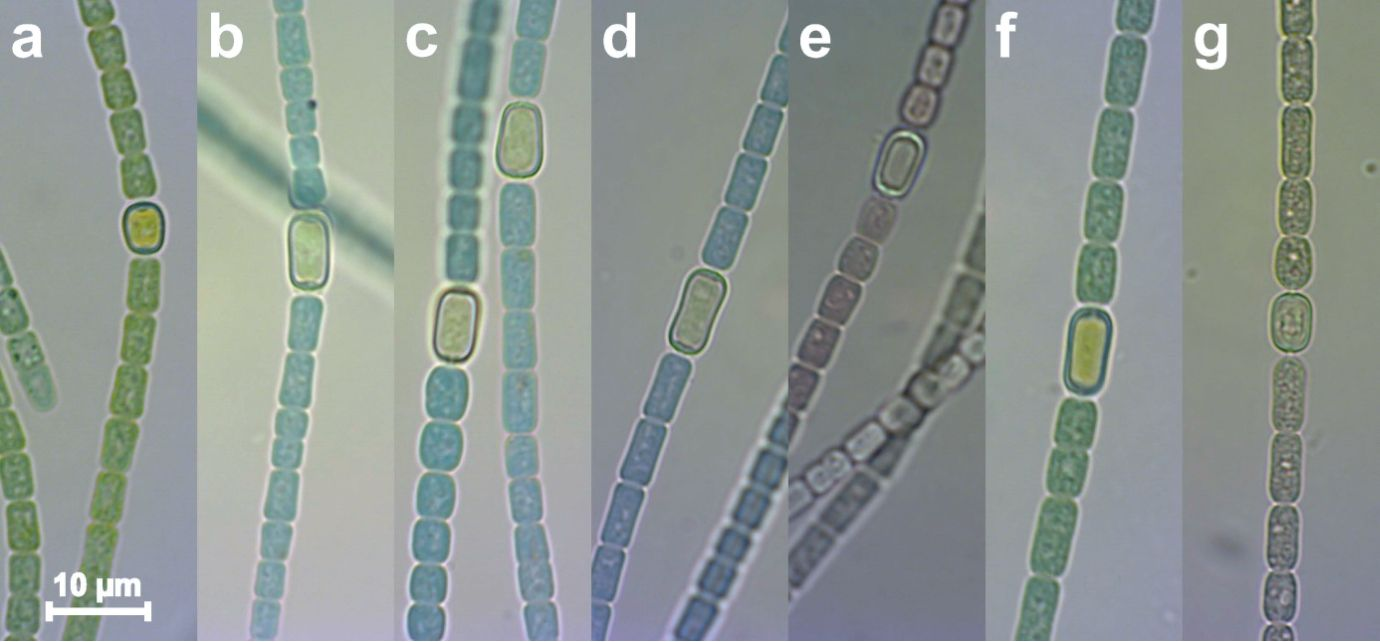
Optical microscopy of *Anabaena cylindrica* cultures developing untreated as reference (a, f) and growing incubated with an overall concentration of 0.8 mM Au^3+^ (b–e). Images were taken before incubation (a), after six hours (b), eleven hours (c) and 24 hours (d) of gold salt incubation. After 190 hours (eight days) (e) no vital cells were found in the incubated culture anymore, while the reference at this time seems to be unaltered (f). Image (g) shows degraded *Anabaena cylindrica* after diluted HCl was added for comparison.

During the experiment samples of the bacterial suspension were taken at selected times and analyzed by light microscopy. Within the period it takes for a drop of the suspension to evaporate, only about eight microscopy images could be taken. Figure 1 shows such images of the *Anabaena cylindrica* organisms before the culture was divided into two parts (Figure 1a), one to grow untreated as a reference and the other to be incubated with an overall concentration of 0.8 mM Au^3+^. Changes of increasing severity can be identified after six hours (Figure 1b), eleven hours (Figure 1c) and 24 hours (Figure 1d) of metal incubation. Some cells have changed their color from green to a mix of blue and brownish, later some dead cells are found. However, the culture stays vital and always a high fraction of organisms with the typical appearance and shape of *Anabaena cylindrica* are found. A sample taken from the solution after more than 190 hours (8 days) of incubation (Figure 1e) revealed only dead organisms. Another sample, which was taken from the untreated reference culture at the same time (Figure 1f), showed its unchanged green color and therefore living organisms with high vitality. The pH value was kept constant in all experiments by adding an appropriate volume of KOH after every drop of the HAuCl_4_ solution added. The last image (Figure 1g) was taken from a culture, which was intentionally exposed to HCl, demonstrating that the color changes in the experiment (Figure 1b–e) are only caused by the gold incubation and not due to a potential pH shift of the added acid.

### X-ray powder diffraction

The crystallinity was proven and the average diameter of the produced nanoparticles was determined by XRD. Washed samples of biomass have been placed on a 25 mm diameter Si(977) disk and dried in ambient air. Spatial inhomogeneity is not affecting the measurement, since the flat sample holder is rotated in the instrument during the measurement by a sample spinner with 1 Hz. The whole area of the disk was filled as completely and as homogeneously as possible. For further details, see also previous section “materials and methods”, Supporting Information File 1 and an earlier study on *Anabaena* sp. [29].

**Figure 2:**
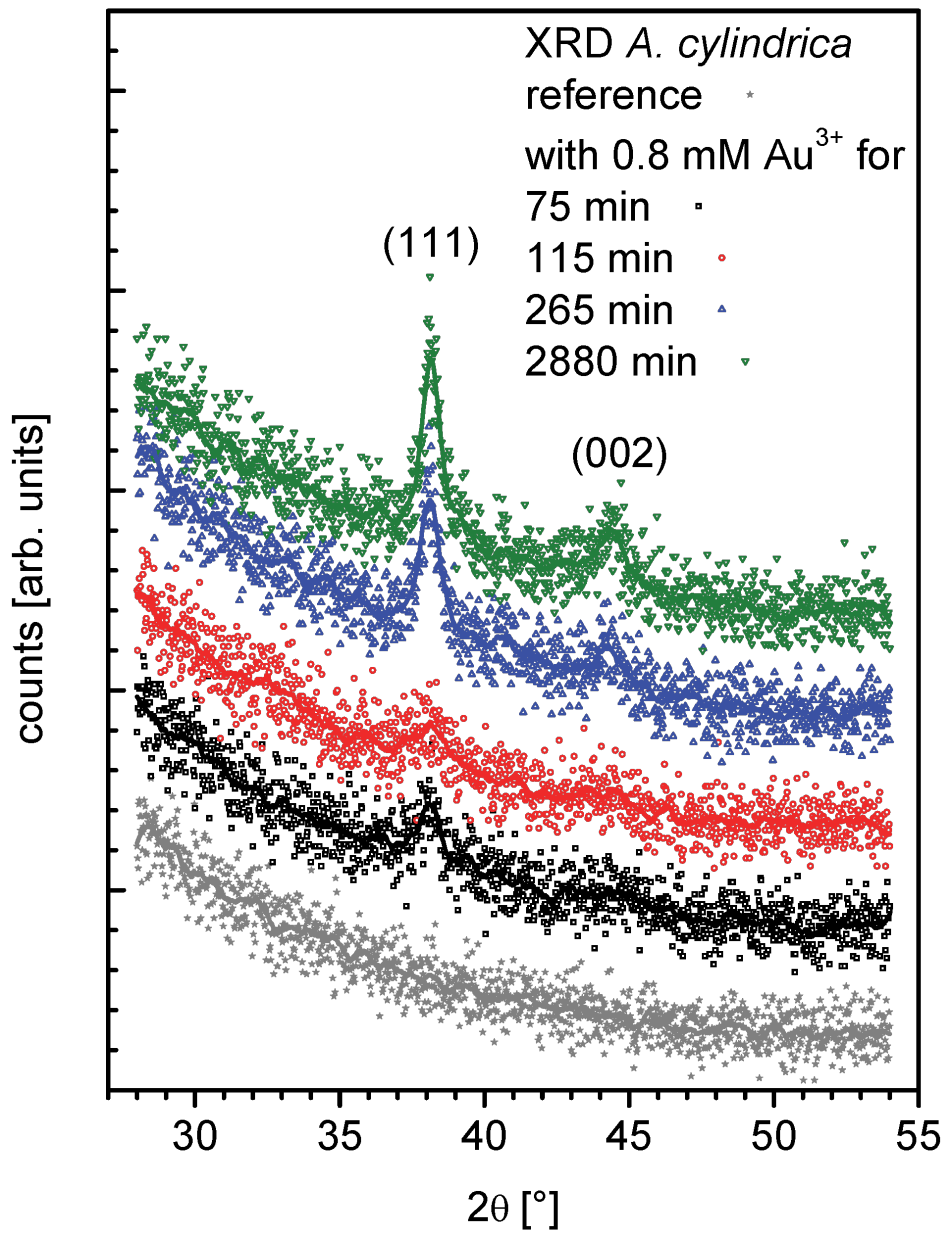
XRD of *Anabaena cylindrica* samples growing incubated with an overall concentration of 0.8 mM Au^3+^ and cultivated without gold salt addition as a reference. Data are stacked for better readability: reference (grey filled stars), sample taken 75 minutes (black open squares), 115 min (red open circles), 265 min (blue open triangles up) and 2880 min (green open triangles down) after the start of gold salt incubation.

XRD of *Anabaena cylindrica* samples were recorded for Bragg angles (2θ) from 28° to 54° with 0.02° per step each 200 seconds. They are plotted stacked in Figure 2 and a smoothened line was added to improve readability. From bottom to top data are shown for a reference without any added gold and therefore no nanoparticle formation (grey filled stars). Further samples were taken 75 minutes (black open squares), 115 min (red open circles), 265 min (blue open triangles up) and 2880 min (green open triangles down) after incubation. At the position of the peaks 111 at 38.1860° and 002 at 44.3833° for gold [58–59] in the four resp. three topmost diffractograms peaks can be clearly identified.

Both peaks have been fitted following the LeBail method [60] using the analysis software JANA2006 [61] to determine their FWHM (full width at half maximum). Within the error margin of the fits the FWHM recorded for the sample with 75 minutes of incubation and that with 115 minutes are the same, giving a good idea about the variation between different cultures resp. samples taken out of the flask of the same culture. However, for samples taken after 265 minutes or longer times the fits show the same peak width.

XRD reveals crystalline regions in the nanoparticles. Further the FWHM of the LeBail fits of the recorded XRD data give information about the size of these regions as an averaged value. If a lot of small nanoparticles are present the average size will be small, even if some have reached their final state. To get information about size distribution a spatially resolving technique like TEM has to be applied, see next section.

The final average size of the formed nanoparticles was found to be around 10 nm and recorded for all the samples taken after more than 265 minutes of incubation in an overall concentration of 0.8 mM Au^3+^. This dimension calculated using the Scherrer-equation [62–63], even the application for particles below 100 nm is complicated by other reasons for diffraction-peak broadening [64], has a quite small standard uncertainty. But it has to be thought of as bound to an uncertainty in the real size of about 1 nm, since the nanoparticles cannot be perfect spheres. This is ensured since the Scherrer-equation gives a difference of about 15% between data taken from the peaks (111) and (002). Nanoparticles are somehow ellipsoidal with an averaged aspect ratio of 1.15. See Supporting Information File 2 for more details.

### Transmission electron microscopy

After the cultures of *Anabaena cylindrica* were incubated with an overall concentration of 0.8 mM Au^3+^, samples were taken after 15 minutes, 9.25 hours and 25.75 hours. These samples were processed for analysis by transmission electron microscopy by separating the supernatant and biomass by centrifugation, washing the biomass and fixing it with glutaraldehyde. TEM was used to determine the spatial distribution of the nanoparticles as well as their sizes.

Although the importance of heterocysts for the biosynthesis is discussed widely in the literature, we have shown in a preceding study a contrary relation for *Anabaena* sp. [29], where vegetative cells have been found to be the cells in which nanoparticles are mainly formed. Nevertheless, the analysis presented here focusses on vegetative cells as well as heterocysts.

The size of the nanoparticles formed inside a cell will mainly be limited by the cell's internal structures, which confines the space for the nanoparticle production [23,29]. This will not be necessarily true for metal nanoparticle formed at the cell-wall at the outside of the cell, but even this constitutes a more restricted location than in a solution. In inorganic systems effects of the confinement on the nanoparticle formation have been found [65–66].

Structures inside the vegetative cells, like the thylakoid membranes, at which photosynthesis takes place, and electron dense spots, most probably representing lipid droplets, are visible by TEM. The extracellular polymeric substances (EPS) on the outside of the vegetative cell are also clearly visible. One prominent feature of heterocysts is the electron dense polar plugs, separating the aerobic environment of the vegetative cells from the anaerobic environment inside the heterocyst. These structures, also called cyanophycin granule (CPG), are sometimes lost during sample preparation for TEM leaving an electron translucent area behind [28]. The second very typical feature, the heterocyst polysaccharide layer (HEP), can be seen as a ragged gray layer of varying thickness around each heterocyst.

We like to stress that the different sizes of cells in an image are not a proof for real differences in cell size but a result of sample preparation. Most likely this is due to different cells being sectioned in different angles, which is not controllable. A barrel for example will show rectangular over elliptic to circular shape only depending of the angle it is cut with respect to its symmetry axis.

**Figure 3:**
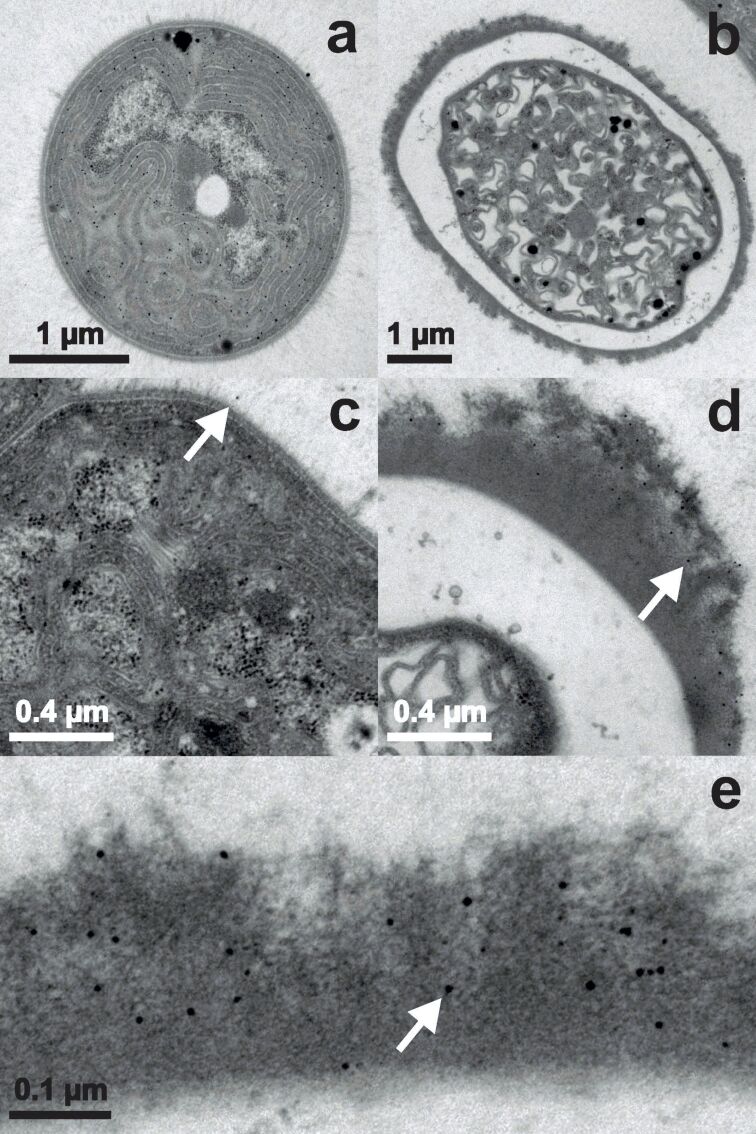
TEM images of *Anabaena cylindrica* incubated with an overall concentration of 0.8 mM Au^3+^ for 15 minutes. Vegetative cells are displayed in panels (a) and (c), heterocysts in panels (b), (d) and (e). The white arrows guide the eye to one already formed nanoparticle in each panel with larger magnification.

Images in Figure 3 are taken from samples incubated with an overall concentration of 0.8 mM Au^3+^ for only 15 minutes. A vegetative cell completely filled with nanoparticles is shown in Figure 3a. Because of the circular appearance the image represents a cross section through a cell. It has to be noted that in only one more vegetative cell of this sequence an equivalent amount of nanoparticles has been found. At the EPS of the vegetative cell (Figure 3c) only a few nanoparticles have been detected (one is indicated by a white arrow). Figure 3b shows a heterocyst with no nanoparticles inside, only in the HEP few and tiny nanoparticles (one is indicated by a white arrow) were observed (Figure 3d). There is a gradient down towards the cell interior with more nanoparticles present at the outside of the HEP. The nanoparticles in the HEP (one is indicated by a white arrow) have a circular shape with a diameter of up to about 10 nm (Figure 3e). As shown in section “Results – X-ray powder diffraction”, the nanoparticles exhibit an ellipsoidal form in average with an aspect ratio of 1.15. Such small differences could not be resolved with the TEM setup used here, mainly because of the electron dense background of the organic material and the pixel size of the recorded images. A pixel represents still more than 1 nm^2^ at the maximum magnification of 85k.

**Figure 4:**
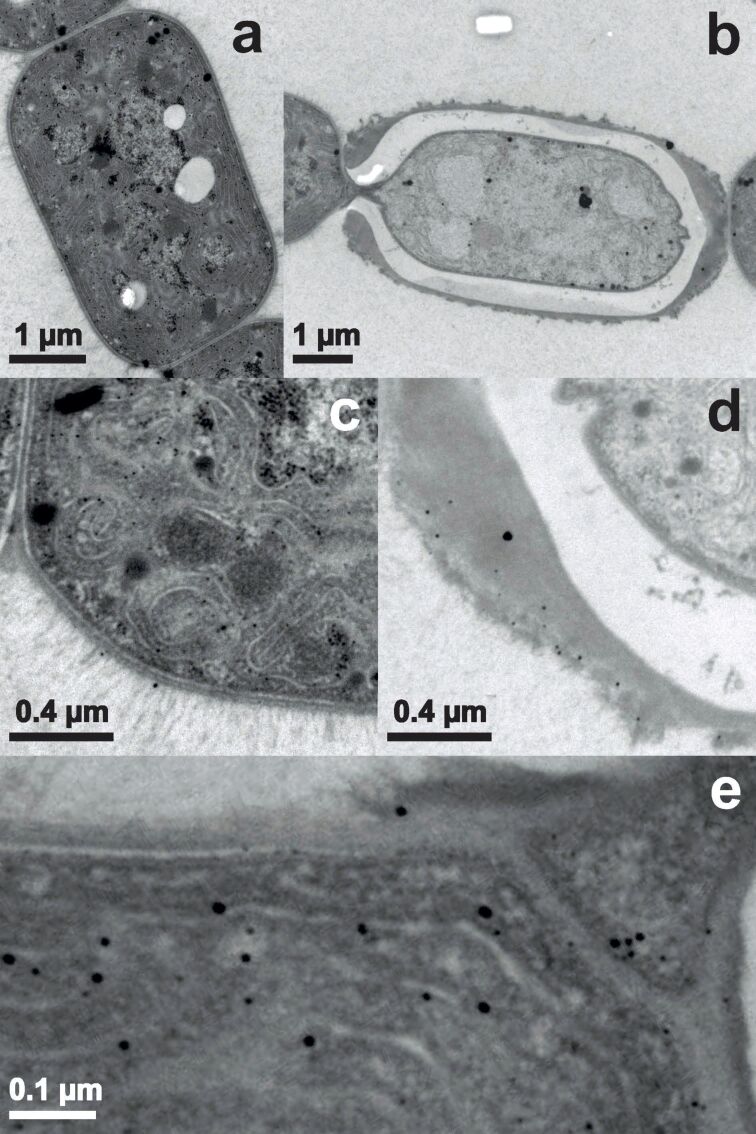
TEM images of *Anabaena cylindrica* incubated with an overall concentration of 0.8 mM Au^3+^ for 9.25 hours. Vegetative cells are displayed in panel (a) and (c), heterocysts in (b), (d) and (e).

Figure 4 represents samples of *Anabaena cylindrica* after 555 minutes of incubation. Figure 4a shows a vegetative cell and Figure 4b a heterocyst. As seen in Figure 4c the vegetative cell is filled with nanoparticles mainly located at the thylakoid membranes and some nanoparticles can be found in the EPS. Figure 4d shows nanoparticles in the HEP. The sizes of the nanoparticles found in the EPS (Figure 4c) and in the HEP (Figure 4d) are comparable. Figure 4e is an enlarged image of the interconnection between the heterocyst shown in Figure 4b and the vegetative cell visible to its left. Near the thylakoid membranes of the vegetative cell nanoparticles with sizes up to 15 nm are found. But also in the polar plug (right upper corner of Figure 4e) nanoparticles are recorded.

**Figure 5:**
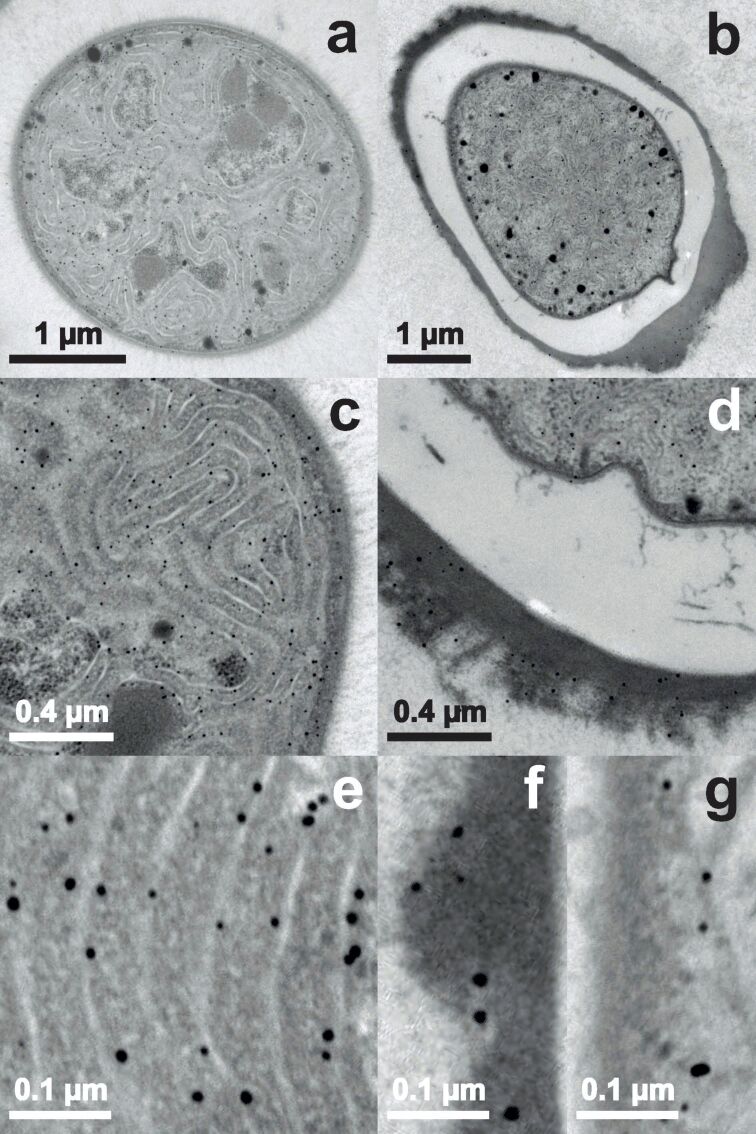
TEM images of *Anabaena cylindrica* incubated with an overall concentration of 0.8 mM Au^3+^ for 25.75 hours. Vegetative cells or parts thereof are displayed in panel (a), (c) and (e), heterocysts or parts thereof in (b), (d), (f) and (g).

Figure 5 shows TEM images from samples incubated for more than one day. In Figure 5a a cross section of a vegetative cell is displayed. The ultrathin section of only about 60 nm thickness contains more than 130 nanoparticles. In the heterocyst shown in Figure 5b nanoparticles still can be found mainly within the HEP, but there are also nanoparticles present within the cell. The size of the formed nanoparticles is similar at all locations. Comparing Figure 5c and Figure 5d, showing the nanoparticles in more detail, it can be seen that the size of the recorded nanoparticles are approximately the same in all vegetative cells, heterocysts and the HEP. From the third row the size distribution and the location of the synthesized nanoparticles can be identified. In vegetative cells (Figure 5e) they are found next to the thylakoid membranes, displaying diameters up to 14 nm and about 10 nm in average. The largest nanoparticle was found in the HEP of a heterocyst (Figure 5f) with a size next to 16 nm. The particles inside the heterocyst (Figure 5g) are comparable in size with those imaged in the vegetative cell (Figure 5e). Overall a nearly circular appearance dominates, while single ones are significantly elongated, compare for example the lower right of Figure 5g.

### Laser-induced breakdown spectroscopy

XRD has revealed the average size of the formed nanoparticles and TEM their spatial distribution in the organism. The total amount of gold uptake was determined utilizing laser-induced breakdown spectroscopy (LIBS). Samples of dried unwashed biomass were placed in a laser beam, which was generating plasma on the sample and the emitted light was analyzed, see section “materials and methods” for more details on this method.

**Figure 6:**
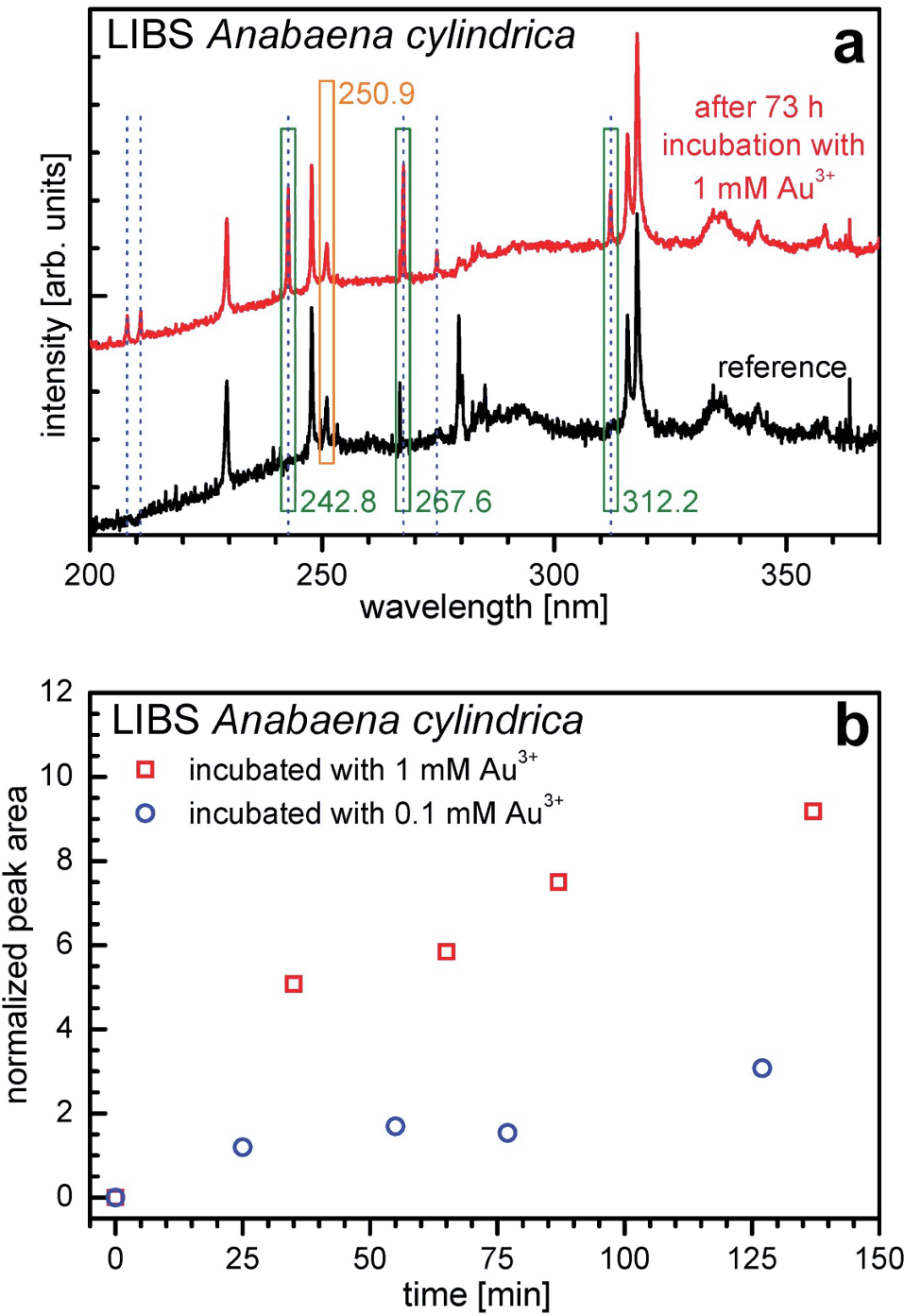
(a) LIBS spectra of dried biomass of *Anabaena cylindrica* (reference, black) and after incubation for 73 hours with an overall concentration of 1 mM Au^3+^ (red). Dotted blue lines indicate positions of characteristic peaks of gold. The green boxes show the peaks originating from gold and the orange box the peak originating from ambient air. The first ones are used as a measure of accumulated amount of gold, the second as an internal reference for the semi-quantitative analysis. (b) Amount of gold recorded in dried unwashed biomass of *Anabaena cylindrica* incubated with an overall concentration of 1 mM (red open squares) resp. 0.1 mM (blue open circles) Au^3+^ over time. This amount of gold is retrieved from the averaged area of the gold indicating peaks (242.8 nm, 267.6 nm, 312.2 nm) divided by the area of one peak from ambient air (250.9 nm).

Figure 6 shows LIBS signals and derived data for samples from *Anabaena cylindrica* incubated with gold ions. In Figure 6a representative LIBS signals are shown. At the bottom a spectrum from a sample of dried untreated *Anabaena cylindrica* biomass (black) is shown. The top curve is measured for dried biomass after the cyanobacteria culture was incubated with 1 mM Au^3+^ for 73 hours (red). The peaks recorded in the reference are characteristic fingerprints of the atoms present in the cyanobacteria and therefore also in the dried biomass as well as the surrounding air above the sample, where the plasma ignites. Comparing both spectra six additional peaks can be identified, marked with vertical dotted blue lines. All elements show peaks at characteristic wavelengths, here those appear additionally, which are specific for gold [55]. Spectral peaks at 208.2 nm, 210.1 nm and 274.8 nm show a low overall intensity. The most prominent peak is found at 267.6 nm, further strong ones are located at 242.8 nm and 312.2 nm. They are depicted in the Figure 6a with green boxes. The analysis is based on these three peaks, which show up at a region with constant background. Since LIBS is very sensitive to variations in samples surface quality and orientation, both affecting the intensity of the induced plasma, an internal reference is needed. At 250.9 nm a quite strong peak is visible, which shows comparable intensity for samples of dried biomass no matter whether incubated with gold or not. The other more intensive peaks at 229.5 nm and 247.7 nm are too much affected by the samples’ composition as to be useful as an internal reference. This peak is depicted in Figure 6a with an orange box and originates from ambient air (oxygen, nitrogen); both elements are always present in the air over the sample [55]. Therefore the peak at 250.9 nm is used as an internal reference.

Each Au peak’s area is a measure for the amount of gold in the biomass. To reduce noise the peak areas integrated individually from the LIBS spectra at 242.8 nm, at 267.6 nm and 312.2 nm are shown as a single averaged value. To handle the problem of non-perfect surfaces and orientation of samples, the averaged value from the gold peaks (242.8 nm, 267.6 nm, 312.2 nm) is divided by the area of the peak at 250.9 nm (ambient air).

In Figure 6b this averaged and normalized value is plotted over time for two experiments, five samples each. One culture was incubated with an overall concentration of 1 mM Au^3+^ (red open squares) and one with 0.1 mM Au^3+^ (blue open circles). All samples shown in Figure 6b were processed in the following manner. First the biomass and the supernatants were separated by centrifugation and then dried directly at ambient air.

The data clearly show two important features. For one thing, larger concentrations of gold ions in the culture result in a higher amount of gold inside the organism. For another thing, as early as 25 minutes after incubation started, even for the low concentration of 0.1 mM, gold can already be determined in the biomass.

## Discussion

The cyanobacterium *Anabaena cylindrica* strain SAG 1403.2 is able to produce nanoparticles out of an aqueous solution with an overall concentration of 0.8 mM Au^3+^. This was seen before for *Anabaena flos-aquae* (ALCP B24) [10] and *Anabaena* sp. (SAG 12.82) [29]. Although *Anabaena flos-aquae* forms gold nanoparticles, it also produces anatoxin-a, see Supporting Information File 1, while *Anabaena* sp. and *Anabaena cylindrica* produce gold nanoparticles without any cyanotoxins (like anatoxin-a) as byproducts [67]. In *Anabaena cylindrica* nucleation seems to take place immediately after starting the incubation, but not at the same rate in all cells of the organism, since first nanoparticles are formed within 15 minutes in some vegetative cells, see Figure 3, while the majority of cells has not produced any at that time. Such fast formation of nanoparticles was not observed in a recent study utilizing *Anabaena* sp. [29]. Leaving these very fast producers aside first nanoparticles are recorded in the HEP of heterocysts, see Figure 3d, afterwards in the EPS of vegetative cells, see Figure 4c, and inside vegetative cells, see Figure 4c, and at last inside heterocysts, see Figure 5d. Finally in all vegetative cells about the same amount of nanoparticles is found, located at the thylakoid membranes.

The main difference between *Anabaena cylindrica* and *Anabaena* sp. (SAG 12.82) [29] is the very fast production of nanoparticles in some vegetative cells of *Anabaena cylindrica*. Beside these extraordinary cells of *Anabaena cylindrica* the time scale of nanoparticle formation is comparable for both cyanobacteria. Within some hours the final size of the biosynthesized nanoparticles is achieved, as recorded by XRD.

**Figure 7:**
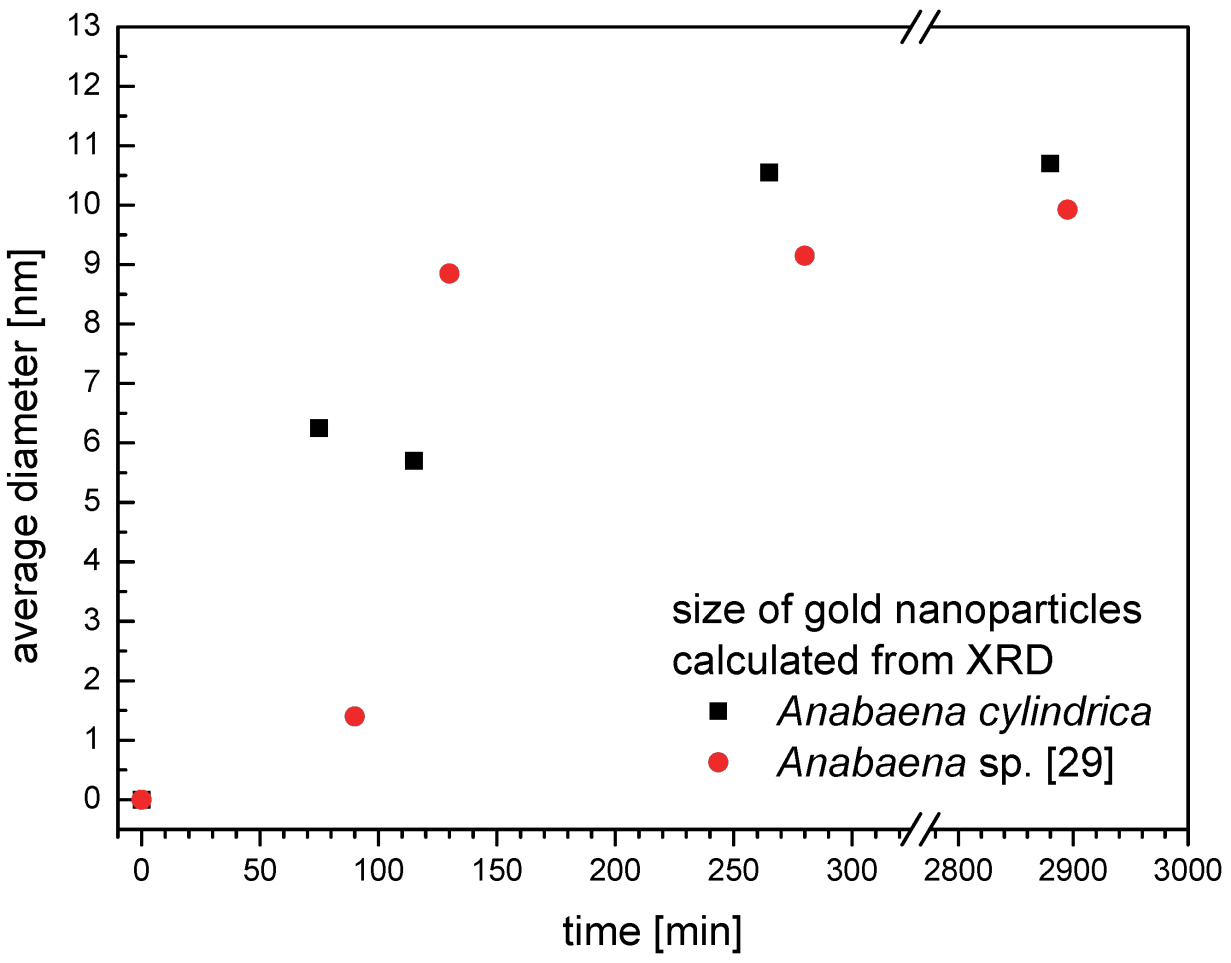
Average size of nanoparticles formed by *Anabaena cylindrica* (black squares) and *Anabaena* sp. [29] (red circles) calculated as average from peak (111) and (002) of the XRD signal over the time of incubation with an overall concentration of 0.8 mM Au^3+^.

In Figure 7 the average size of the gold nanoparticles is shown over the time of incubation for *Anabaena cylindrica* (black squares) and *Anabaena* sp. (red circles) [29]. Both organisms were incubated with an overall concentration of 0.8 mM Au^3+^. As described in section “Results – X-ray powder diffraction” the nanoparticles cannot be perfect spherical, since the sizes calculated from the peaks (111) and (002) are always slightly different. Therefore here the mean from both values is used as a measure of average size. After some hours this size is not increasing anymore, compare especially data for more than one day of incubation.

Over the development of the nanoparticles the average size is slightly larger for *Anabaena cylindrica* than for *Anabaena* sp. as calculated from XRD measurements. The former species reached the final average size of 10 nm latest within four hours, the latter 9 nm latest within two hours. All these calculated averages are connected to an uncertainty of about 1 nm in reality. Therefore the recorded differences in the final average size do not seem to be significant.

Within the error margin this difference between species is smaller than that between aliquots of the same species. As example for the variation between samples within the same species compare TEM images for *Anabaena cylindrica* for 75 (Figure 3) and 115 minutes (Figure 4). For two different species take as example the data of more than 2800 minutes incubation from XRD as shown in Figure 7.

Interestingly there is a more prominent difference to *Anabaena flos-aquae*, where the first nanoparticles have been recorded after five minutes [23]. It has to be tested in detail whether this is due to a different behavior of species or the environmental parameters chosen in this study. Therefore, the dependency on all the environmental parameters specified in the experimental section has to be determined: If the interspecies difference is significant, a different answer on changed parameters, e.g., concentration, temperature and pH, is expected. Furthermore in all TEM images the gold nanoparticles appear quite spherical, while the XRD measurements reveal a slightly ellipsoidal shape with an aspect ratio of 1.15. It is known from literature that environmental parameters like pH [31–32,68–70], temperature [17,40], oxidation state of the metal atoms [71], concentration of metal ions [72] and counter ions [73] affect the morphology and size of the nanoparticles formed by various organisms.

LIBS experiments have confirmed the observation of XRD and TEM that nanoparticle formation takes place in the first few hours after exposure to Au^3+^. As seen in Figure 6 the amount of gold is continuously increasing over the first two hours for *Anabaena cylindrica*. The time-dependent development seems to be the same for a ten times smaller concentration, see Figure 6b. Since LIBS utilizes the light from a micro-plasma generated by a laser beam on the sample’s surface, all information about the chemical state (oxidation state, neighborhood) in the sample (biomass) is lost, only the presence of specific elements can be proven. The amount of gold detected is not necessarily exclusively the mass of the produced nanoparticles as detected with XRD or TEM. Besides, LIBS will also detect remaining Au^3+^ ions or clusters as well as tiny nanoparticles below the detection limit of both other techniques. The XRD method is limited by the need of crystallinity. Smaller crystallites result in broader peaks, compare Scherrer-equation [62–63]. Since those broad peaks vanish in the noise of the background at some point, the lower limit of detection are some nanometers in one dimension. The instrumental setup used here was able to detect nanoparticles with an average size of as low as 1.5 nm as shown in Figure 7 (*Anabaena* sp. 95 minutes), but calculated sizes below 2 nm suffer, because of the above mentioned problems, from a large uncertainty, e.g., [64] even states that the methods should not be used for particles below 100 nm.

The spatial resolution of TEM is limited by its electron beam energy and even more by the pixel size of the digital image. In the setup used here, a pixel corresponds for the maximum magnification of 85k to 1.15 nm × 1.15 nm. Nanoparticles with a diameter of less than 4 nm are quite hard to visualize in ultrathin sections due to the electron dense background caused by the heavy metal stained biomass. Such tiny, nearly spherical nanoparticles have already a volume of more than 30 nm^3^ (1 nm^3^ crystalline gold contains around 60 gold atoms).

From TEM images an average nanoparticle concentration inside the cells can be determined just by counting the nanoparticles and calculating the volume of the ultrathin section used as TEM sample (about 60 nm thick). For the final state after one day about 800 nanoparticles are found in one cubic micrometer of a heterocyst and about 1500 nanoparticles in the same volume of a vegetative cell. The average sizes of the nanoparticles of 10 nm as determined by XRD equal about 3·10^4^ gold atoms per nanoparticle (about 5·10^−20^ mol). The average molar concentration of detectable gold nanoparticles can be estimated with 40 mM in heterocysts and 78 mM in vegetative cells. Even within the large errors of such an approximation, the gold concentration inside both cells is finally much higher than outside, where it was initially 0.8 mM.

**Figure 8:**
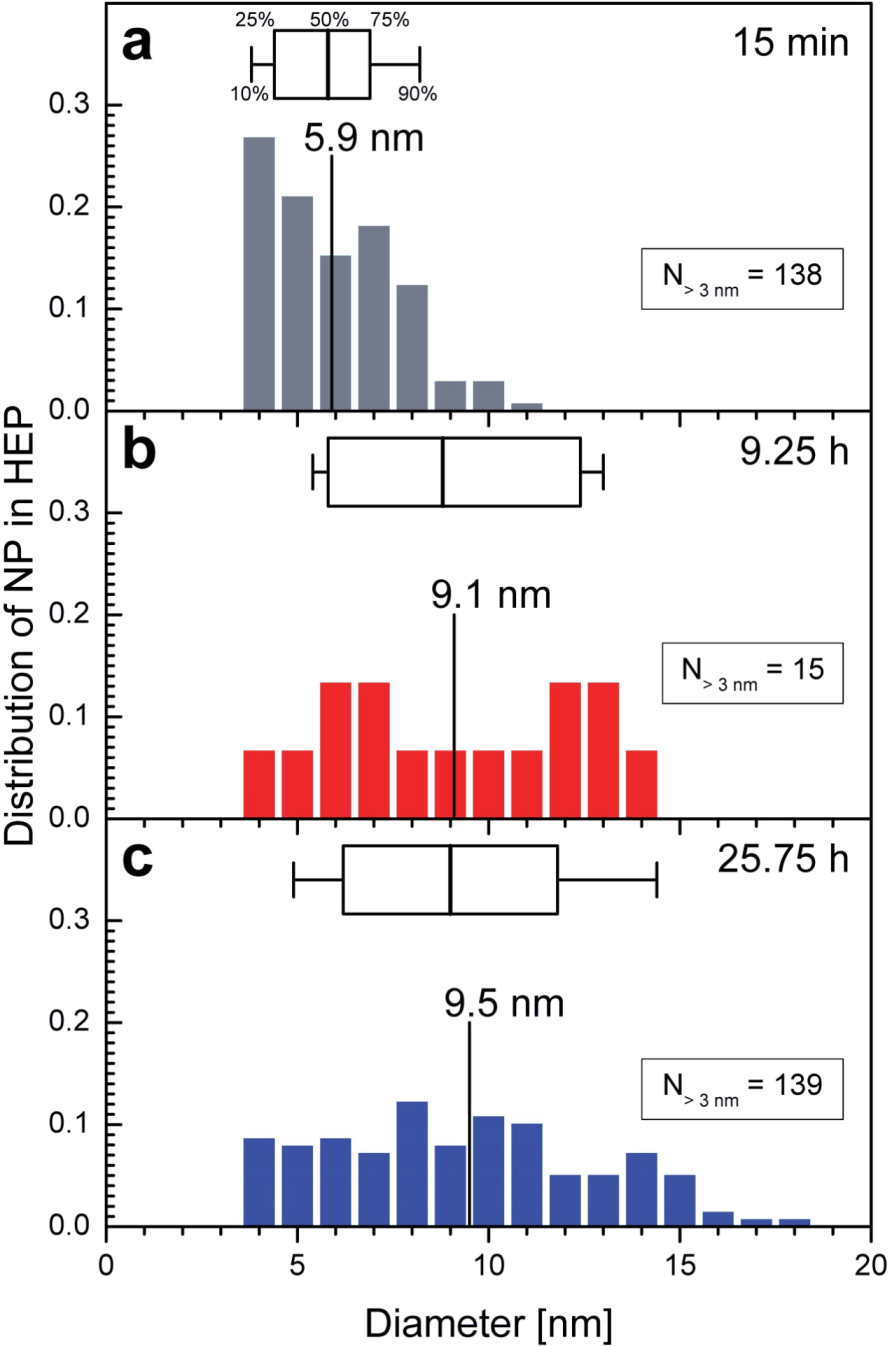
Distribution of the diameter of nanoparticles formed in the heterocyst polysaccharide layer (HEP) of *Anabaena cylindrica* as obtained from TEM images with 50k. Distributions of nanoparticles larger than the reliable detection limit of 3 nm are shown (a) after 15 minutes, (b) after 9.25 hours and (c) after 25.75 hours of incubation with an overall concentration of 0.8 mM Au^3+^. In each panel the mean size (vertical labeled line) is shown as well as a box plot (25% | 50% | 75%) with whiskers for 10% and 90%.

Since XRD detects only the average size of the formed nanoparticles, the size of individual nanoparticle was determined from TEM images with a magnification of 50k and above to reveal the size distribution of the nanoparticles in specific regions of the organism using an image processing tool. Only nanoparticles larger than 3 nm are included, see Supporting Information File 2 for details. Figure 8 shows the distribution of nanoparticles’ diameter in the HEP for the three tested times. The size distribution of 138 nanoparticles found in the HEP after 15 minutes of incubation is shown in Figure 8a. The averaged diameter calculated from this data is 5.9 nm. Figure 8b shows the size distribution after 9.25 hours of incubation. Based on 15 nanoparticles the average is 9.1 nm. Figure 8c contains the analogous distribution for 139 nanoparticles found in the HEP after 25.75 hours of incubation with an average diameter of 9.5 nm. The size distributions of the nanoparticles in the HEP are wide at all times, but a clear shift towards larger nanoparticles can be seen over time. The small increase in diameter after 9.25 hours (Figure 8b) and 25.75 hours (Figure 8c) is not significant.

**Figure 9:**
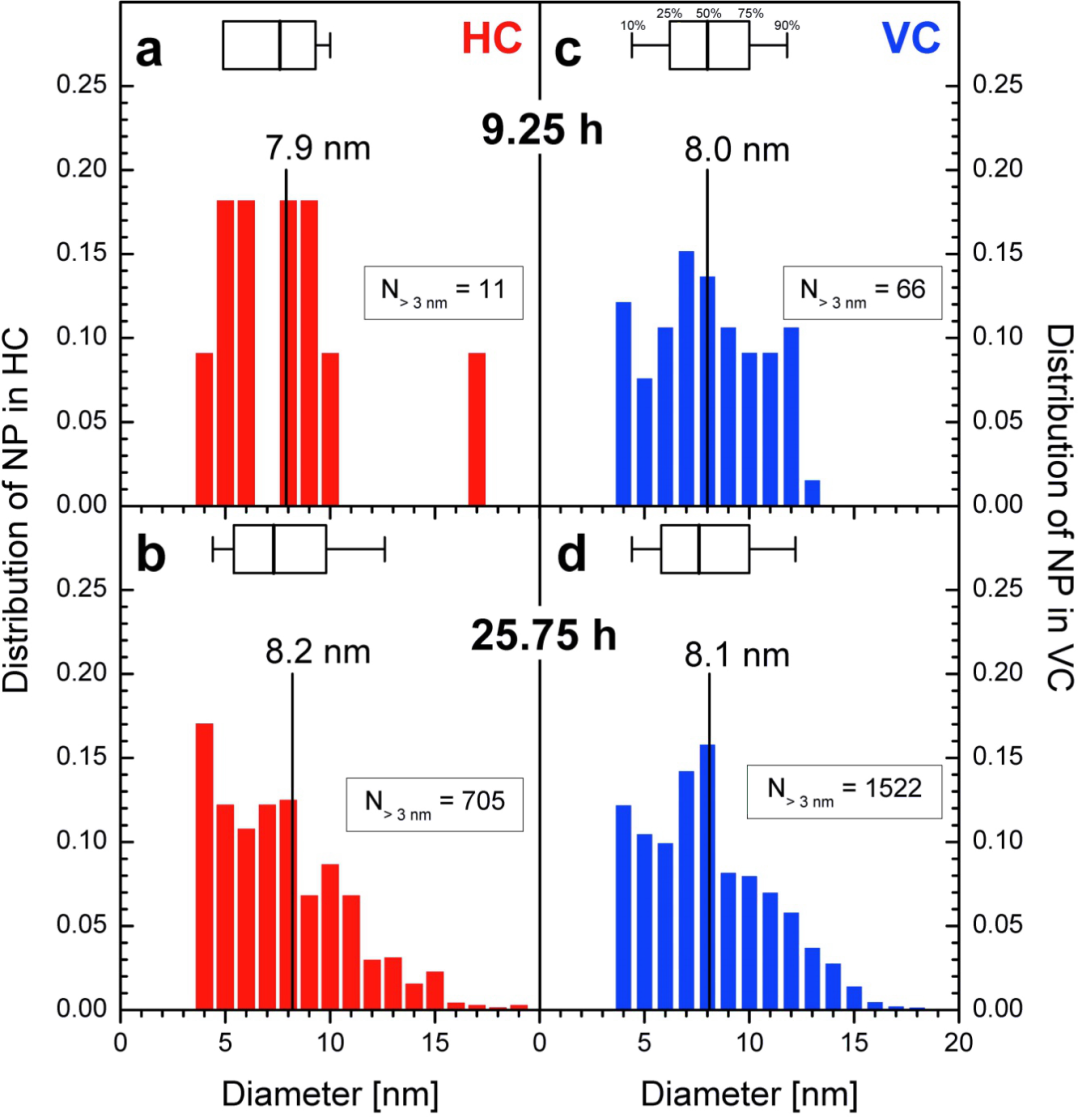
Distribution of the diameter of nanoparticles formed in the heterocysts (HC) and vegetative cells (VC) of *Anabaena cylindrica* as obtained from TEM images with 50k. Distributions of nanoparticles larger than the reliable detection limit of 3 nm are shown in the left column for HC (a, b) and in the right one for VC (c, d) after 9.25 hours (a, c) and 25.75 hours (b, d) of incubation with an overall concentration of 0.8 mM Au^3+^. In each panel the mean size (vertical labeled line) is shown as well as a box plot (25% | 50% | 75%) with whiskers for 10% and 90%.

Figure 9 shows the distribution of nanoparticles’ diameter in the HC and VC for two periods of incubation. In TEM images with a magnification of 50k or more from samples after 15 minutes incubation no nanoparticles were found in HC and VC. Figure 3a has a too small magnification to be analyzed in this way. The left column shows the data for the HC (a, b), the right for VC (c, d). In the top row the data are printed for samples after 9.25 hours of incubation and in the lower after 25.75 hours. Only 11 nanoparticles were recorded within HC with an average diameter of 7.9 nm resp. median 7.6 nm (Figure 9a), while 705 nanoparticles with 8.2 nm (average resp. 7.3 nm median) in samples incubated for 25.75 hours (Figure 9b). In images showing VC after 9.25 hours of incubation 66 nanoparticles with 8.0 nm diameter in average and median (Figure 9c) were recorded. 1522 nanoparticles with 8.1 nm (average resp. 7.6 nm median) were found in TEM images taken from samples after 25.75 hours (Figure 9d). The distribution becomes wider with time as seen for the HEP in Figure 8 and the differences in the calculated diameters are not significant.

XRD has shown that the average size reaches its final value after four hours, so it is expected that the calculated diameters stay the same between 9.25 hours and 25.75 hours as seen for the HEP (Figure 8b,c), HC (Figure 9a,c) and VC (Figure 9b,d). From XRD an average size of 10 nm is determined, see Figure 7, with image processing of TEM images 8 nm (VC, HC) to 9 nm (HEP). With respect to the uncertainties of the methods used the determined average diameter of the nanoparticles is in good agreement the same. XRD preferably detects large nanoparticles since crystallinity is needed and image processing of TEM images is very likely to detect more small ones because of the electron dense and structured background. For a more detailed study better contrasted TEM images with higher resolution will be needed.

As described above, after two to four hours the detectable nanoparticles (XRD, TEM) have reached a plateau in size. Further growth might occur at longer times due to ripening or uptake of remaining gold ions. These processes are outside the scope of the study presented here, since the organisms have died within eight days and without self-reproducing bioreactors no real in vivo biosynthesis is possible anymore.

The amount of biomass for the experiment with *Anabaena cylindrica* presented here and for the experiment with *Anabaena* sp. [29] was significantly different, more than six gram per 150 mL culture volume in the first and less than two gram per 150 mL culture volume in the second. But the speed of nanoparticle formation as well as the finally reached average size is nearly the same, see Figure 7, surprisingly no evidence for a relating effect between biomass and offered amount of metal ions at constant container size was found.

But more interesting is the fact that for both cyanobacteria, *Anabaena cylindrica* and *Anabaena* sp. [29], the heterocysts are not the favorite place for nanoparticle formation. Shortly after the incubation the first nanoparticles were found within the HEP of the heterocysts, but at least for *Anabaena cylindrica* even after only 15 minutes some vegetative cells are already completely filled with nanoparticles, see Figure 3. The final particle size is reached after about four hours, see Figure 7 from XRD data, and probed with TEM after one day. The number of nanoparticles inside the heterocysts is still smaller than that in vegetative cells, see Figure 6. In vegetative cells the gold nanoparticles are located at the thylakoid membranes and should not be changed anymore by ongoing biosynthesis. This study emphasizes the finding of an earlier study utilizing *Anabaena* sp. [29], that heterocysts are not as important for nanoparticle formation as described for *Anabaena flos-aquae* [23], since more than 10 vegetative cells come on one heterocyst.

*Anabaena cylindrica* is therefore a promising bioreactor for biosynthesis of nanoparticles, because no anatoxin-a is produced and the process of biosynthesis is therefore non-hazardous.

As the number of nanoparticles in the HEP of the heterocysts is decreasing with time, compare Figure 3 and Figure 4, some nanoparticles formed inside the HEP have to be released into the supernatant over time, since the number of nanoparticles inside the heterocysts is not increasing in the same manner. This release may explain the coloring of the culture medium as seen in Figure 1. Such a coloring was also observed by Brayner et al. for *Anabaena flos-aquae* [23] as well as in our study for *Anabaena* sp. [29]. These particles can neither be found in XRD or TEM since the samples are washed during preparation to ensure only the presence of biomass bound nanoparticles, see “Materials and Methods” section for details. LIBS in the supernatants can proof this, but this is a challenging task since humidity makes measurements complicated for this method.

## Conclusion

The cyanobacterium *Anabaena cylindrica* can be employed as a bioreactor for the in vivo biosynthesis of gold nanoparticles from aqueous gold solutions in an environmentally compatible way, especially since it does not produce anatoxin-a. Utilizing LIBS it was shown that the uptake of gold in the biomass is nearly linear within the first two hours of incubation and takes place for as low molar concentrations as 0.1 mM. First nanoparticles form within some minutes in the HEP. Later most nanoparticles are found inside vegetative cells at the thylakoid membranes and heterocysts. Within four hours of incubation the average size of nanoparticles reaches a constant value between 8 and 10 nm. Even though the average size in XRD is constant it could not be excluded that small particles have formed, since they would not have been visible in TEM. In all images a wide spread distribution of nanoparticles’ diameter was detected. The time-dependent growth of nanoparticles and the distribution of their size were examined for the first time on a time scale of hours up to at least two days of incubation. TEM reveals a large variation between individual cells and most nanoparticles are found in vegetative cells and not in the heterocysts as described in literature before, especially for *Anabaena flos-aquae* in [23].

## Supporting Information

File 1 Cultures and preparation in the study on cyanobacteria *Anabaena cylindrica* as self-reproducing bioreactors.

File 2 Analyzing methods in the study on cyanobacteria *Anabaena cylindrica* as self-reproducing bioreactors.
