# Supplementary material for: Time-dependent growth of crystalline Au0-nanoparticles in cyanobacteria as self-reproducing bioreactors: 2. Anabaena cylindrica
Source: Beilstein J Nanotechnol. 2016 Mar 2;7:312–27. doi: 10.3762/bjnano.7.30 (PMC4901539; doi:10.3762/bjnano.7.30)
Supplement: File 1 — Cultures and preparation in the study on cyanobacteria Anabaena cylindrica as self-reproducing bioreactors. [file Beilstein_J_Nanotechnol-07-312-s001.pdf]

Supporting Information

for

Time-dependent growth of crystalline Au^0-nanoparticles in cyanobacteria as self-reproducing bioreactors: 2. *Anabaena cylindrica*

Liz M. Rösken[1], Felix Cappel[1], Susanne Körsten[1], Christian B. Fischer[1], Andreas Schönleber[2], Sander van Smaalen[2], Stefan Geimer[3], Christian Beresko[4], Georg Ankerhold[4] and Stefan Wehner*[1,§]

Address: [1]Universität Koblenz-Landau, Institut für Integrierte Naturwissenschaften, Abteilung Physik, 56070 Koblenz, Germany, [2]Universität Bayreuth, Lehrstuhl für Kristallographie, 95440 Bayreuth, Germany, [3]Universität Bayreuth, Zellbiologie / Elektronenmikroskopie, 95440 Bayreuth, Germany and [4]Hochschule Koblenz, RheinAhrCampus Remagen, Optics and Laser Engineering, 53424 Remagen, Germany

Email: Liz M. Rösken - roesken@uni-koblenz.de; Felix Cappel - fcappel@uni-koblenz.de; Susanne Körsten - skoersten@uni-koblenz.de; Christian B. Fischer - chrbfischer@uni-koblenz.de; Andreas Schönleber - andreas.schoenleber@uni-bayreuth.de; Sander van Smaalen - smash@uni-bayreuth.de; Stefan Geimer - stefan.geimer@uni-bayreuth.de; Christian Beresko - beresko@hs-koblenz.de; Georg Ankerhold - ankerhold@hs-koblenz.de; Stefan Wehner - wehner@uni-koblenz.de
* Corresponding author

[§] phone: +49-261-287-2341 fax: +49-261-287-100-2341

Cultures and Preparation in the study on cyanobacteria *Anabaena cylindrica* as self-reproducing bioreactors

Cultures

Throughout this study cyanobacterium *Anabaena cylindrica* strain 1403.2 (Algensammlung Göttingen, SAG) was tested on its ability as a bioreactor. The most prominent type *Anabaena flos-aquae* was left out in an early stage, since a small but measureable amount of anatoxin-a was detected in these cultures following the method described in [S1 = 66].

Table S1: Concentration of anatoxin-a in different algae cultures.

	**Anatoxin-a**
Anabaena spec. (n=3)	< LOQ
Anabaena flos-aquae (n=3)	>50 µg/L
Anabaena cylindrica (n=6)	< LOQ

Limit of quantification (LOQ): 0.02 µg/L (anatoxin a);
n: number of investigated algae samples

The concentration of anatoxin-a exceeded the linear working range of the analytical method in all investigated *Anabaena flos-aquae* samples. Therefore, the concentration of anatoxin-a was set to greater than 50 µg/L. The concentration of cylindrospermopsin (0.02 µg/L), nodularin-R (0.01 µg/L), microcystin-LA (0.01 µg/L), microcystin-LF (0.03 µg/L), microcystin-LY (0.01 µg/L), microcystin-RR (0.01 µg/L), microcystin-YR (0.01 µg/L), domoic acid (0.06 µg/L) and okadaic acid (0.06 µg/L) were below the limit of quantification (LOQ enclosed in parentheses).

The nanoparticle producing cultures have been grown for a period until the end of logarithmic phase and the begin of the stationary phase is reached. For *Anabaena cylindrica* this has been twelve weeks under the above specified conditions. At this point the cultures have been split, one half for incubation, the other as reference.

Preparation for XRD analysis

Samples for XRD have been prepared according to the following protocol. After a first centrifugation step with 14,000 rpm (equals 16,000*g* for the Eppendorf centrifuge 5154 used) for 15 minutes, the supernatant was separated from the biomass. The first one was used for UV–vis spectroscopy. The biomass was washed by resuspension in bidistilled water and a second centrifugation step (15 min, 14,000 rpm, 16,000*g*), to ensure the complete removal of all gold ions and clusters not located inside the cells.

The washing water was discarded and the biomass was resuspended in bidistilled water. At this stage the process was interrupted, and the resuspended biomass stored in a deep freezer until it was spread as thin and homogeneously as possible on the homemade sample holders. Therefore the biomass was separated from the added water by centrifugation again.

Preparation for TEM analysis

Samples for TEM have been prepared following standard procedures. First the biomass was separated in a centrifugation step (15 min, Eppendorf centrifuge 5154, 14,000 rpm equal 16,000*g*) from the supernatant, which was used for UV-vis spectroscopy. Cells were incubated in fixation buffer (0.1 M HEPES, 4 mM $CaCl_2$, 2.5% glutaraldehyde, pH 7.2) at room temperature for 30 minutes and, after replacing the fixative with fresh one, overnight at 4 °C. Latest after fixation with glutaraldehyde the process of nanoparticle production process was interrupted. After one wash of ten minutes in distilled water the samples were osmicated in 2% osmium tetroxide (in distilled water) for 120 minutes at 4 °C. From this stage another centrifuge was used (Beckmann Coulter Microfuge 11, 13,000 rpm equal 8,500*g*). After three washes of 20 minutes each in distilled water the samples were embedded in 1% Difco™ Agar noble (Becton, Dickinson and Company, Sparks, MD, U.S.A.), dehydrated using increasing concentrations of ethanol and embedded in glycid ether 100 (formerly Epon 812; Serva, Heidelberg, Germany) with propylene oxide as intermediate solvent following standard procedures. Polymerization was carried out for 48 hours at 65 °C. Ultrathin sections of 50-60 nm were obtained using an Ultracut EM UC6 ultramicrotome (Leica Microsystems, Wetzlar, Germany) with a diamond knife (type ultra 45°, Diatome, Biel, Switzerland), collected on Pioloform-coated copper slot grids

(Plano, Wetzlar, Germany) and stained with uranyl acetate and lead citrate [S2]. Samples were analyzed using an EM 902A (Carl Zeiss, Oberkochen, Germany) transmission electron microscope operated at 80 kV. Micrographs were taken using a 1,350 x 1,050 pixel Erlangenshen ES500W CCD camera (Gatan, Pleasanton, CA, USA) and Digital Micrograph software (version 1.70.16, Gatan).

References

[S1 = 67] Schlüsener, M.; Beel, R.; Bröder, K; Luft, A., Ternes, T., Ed. *Klimabedingt verändertes Muster organischer Schadstoffe in Bundeswasserstraßen. Schlussbericht KLIWAS-Projekt 5.04/3.07. KLIWAS-51/2014,* BfG, Koblenz, **2014**.

[S2] Reynolds, E. S. *J Cell Biology* **1963**, *17*, 208–212.
