# Supplementary material for: Time-dependent growth of crystalline Au0-nanoparticles in cyanobacteria as self-reproducing bioreactors: 2. Anabaena cylindrica
Source: Beilstein J Nanotechnol. 2016 Mar 2;7:312–27. doi: 10.3762/bjnano.7.30 (PMC4901539; doi:10.3762/bjnano.7.30)
Supplement: File 2 — Analyzing methods in the study on cyanobacteria Anabaena cylindrica as self-reproducing bioreactors. [file Beilstein_J_Nanotechnol-07-312-s002.pdf]

Supporting Information

for

Time-dependent growth of crystalline Au^0-nanoparticles in cyanobacteria as self-reproducing bioreactors: 2. *Anabaena cylindrica*

Liz M. Rösken[1], Felix Cappel[1], Susanne Körsten[1], Christian B. Fischer[1], Andreas Schönleber[2], Sander van Smaalen[2], Stefan Geimer[3], Christian Beresko[4], Georg Ankerhold[4] and Stefan Wehner*[1,§]

Address: [1]Universität Koblenz-Landau, Institut für Integrierte Naturwissenschaften, Abteilung Physik, 56070 Koblenz, Germany, [2]Universität Bayreuth, Lehrstuhl für Kristallographie, 95440 Bayreuth, Germany, [3]Universität Bayreuth, Zellbiologie / Elektronenmikroskopie, 95440 Bayreuth, Germany and [4]Hochschule Koblenz, RheinAhrCampus Remagen, Optics and Laser Engineering, 53424 Remagen, Germany

Email: Liz M. Rösken - roesken@uni-koblenz.de; Felix Cappel - fcappel@uni-koblenz.de; Susanne Körsten - skoersten@uni-koblenz.de; Christian B. Fischer - chrbfischer@uni-koblenz.de; Andreas Schönleber - andreas.schoenleber@uni-bayreuth.de; Sander van Smaalen - smash@uni-bayreuth.de; Stefan Geimer - stefan.geimer@uni-bayreuth.de; Christian Beresko - beresko@hs-koblenz.de; Georg Ankerhold - ankerhold@hs-koblenz.de; Stefan Wehner - wehner@uni-koblenz.de
* Corresponding author

[§] phone: +49-261-287-2341 fax: +49-261-287-100-2341

Analyzing methods in the study on cyanobacteria *Anabaena cylindrica* as self-reproducing bioreactors

Microscopy

In the beginning of the experiment fluorescence microscopy (Axioimager M2, Zeiss) and optical microscopy (Axioskop 50, Zeiss) were used. The condition of the cultures has been monitored by red fluorescence.

A fluorescence microscopy image is depicted in **Figure S1**. An *Anabaena cylindrica* culture grown for twelve weeks in modified BBM shows a significant red fluorescence in the vegetative cells because of chlorophyll a. The large cell without red fluorescence is a heterocyst, the smaller ones with significant red fluorescence are vegetative cells.

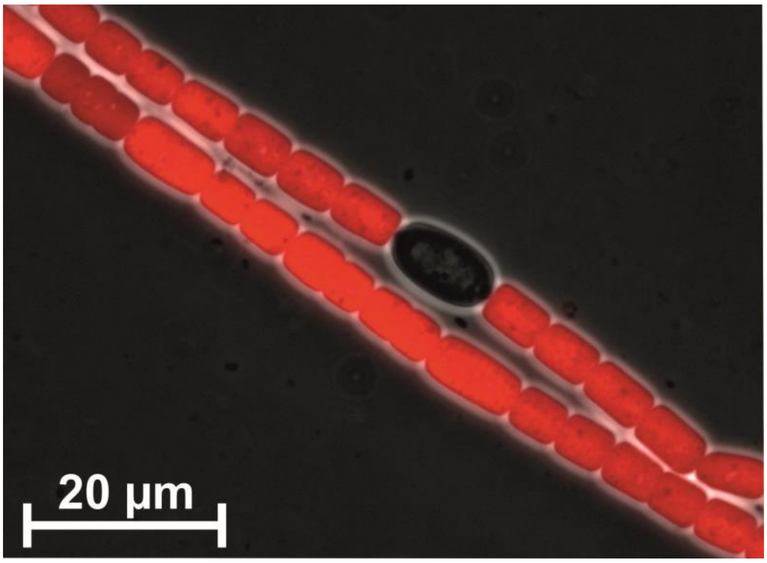

Figure S1: Fluorescence microscopy: *Anabaena cylindrica* grown for twelve weeks in modified BBM.

Morphologically *Anabaena cylindrica* consists of long chains of up to 80 cells, coiled together in rather large conglomerates and surrounded by a matrix of EPS. The chains are separated by heterocysts in segments containing up to 20 vegetative cells. In the light microscope this cell type can easily be distinguished from vegetative cells, since heterocysts are comparatively large, appear to be empty, have a thick cell wall and polar plugs [S3, S4, S5 = 28]. Freshly formed heterocysts in contrast to fully evolved ones might still contain small amounts of chlorophyll a and their shape is more similar to vegetative cells. Fully evolved heterocysts do not contain any chlorophyll a [S6 = 38, S7 = 41].

X-ray powder diffraction

X-ray powder diffraction (XRD) is applied to determine the average size of the formed nanoparticles. Therefore the peaks, showing up in Figure 2 of the main manuscript, are fitted following the LeBail method [S8 = 60; S9 = 61] first and the so determined FWHM is converted using the Scherrer-equation into a characteristic size of the crystallites [S10 = 62, S11 = 63].

The normalized fitted data are shown in **Figure S2**. In the left panel data from the peak (111) are plotted, in the right panel those for (002). The later ones contain less data points, since the angular position is larger and the signal intensity is less. Therefore the fits are as expected not as good as for the peak (111). Within the error margin the fits for 75 minutes incubation (black open squares) and 115 min (red open circles) are the same, giving a good idea about the variation between different cultures resp. samples taken from the same culture at the same time. However, latest after 265 minutes (blue open triangles up) the final average size of 10 nm is reached.

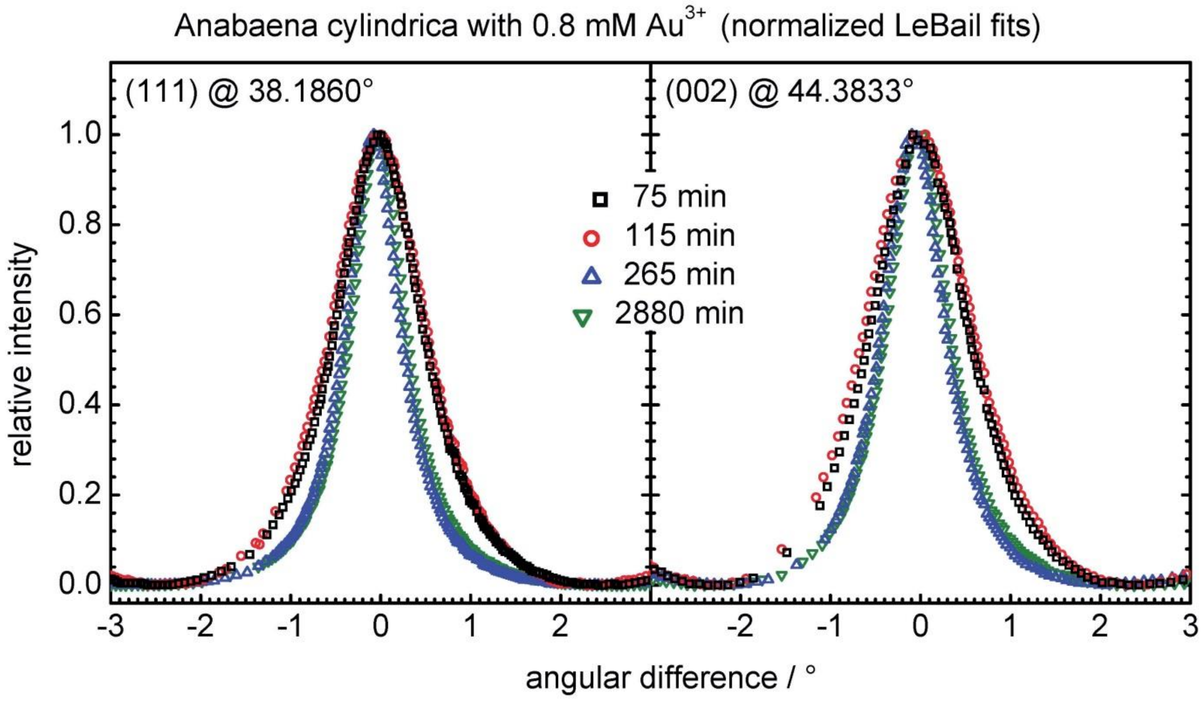

Figure S2: XRD data of *Anabaena cylindrica* samples incubated in an overall concentration of 0.8 mM Au^{3+} analyzed with the LeBail method [S8 = 60, S9 = 61]. Normalized fitted signals from the peak (111) (left) and (002) (right) for incubation times of 75 min (black open squares), 115 min (red open circles), 265 min (blue open triangles up) and 2880 min (green open triangles down) are shown.

$$L = \frac{K\lambda}{\beta \cos\left(\frac{2\theta}{2}\right)} \qquad \text{(S1)}$$

Using the Scherrer-equation (Equation S1) [S10 = 62, S11 = 63] the average size of the crystallites can be calculated. Therein K is the Scherrer constant and equals 0.8290 for spherical crystallites [S11 = 63]. β is the FWHM of the recorded peaks in radiant (LeBail method), 2θ the position of the diffraction signal (Bragg angle) and λ the wavelength used (0.1540598 nm, Cu $K_{\alpha 1}$).

The average size of gold nanoparticles found in *Anabaena cylindrica* samples is given in **Figure S3** as a function of time. As seen in the lower panel the diameter calculated from peaks (111) and (002) differs. This shows an imperfect spherical shape of the nanoparticles [S11 = 63]. Assuming an ellipsoid the aspect ratio of the formed gold nanoparticles is with a value of about 1.15 in average, see upper panel in Figure S3.

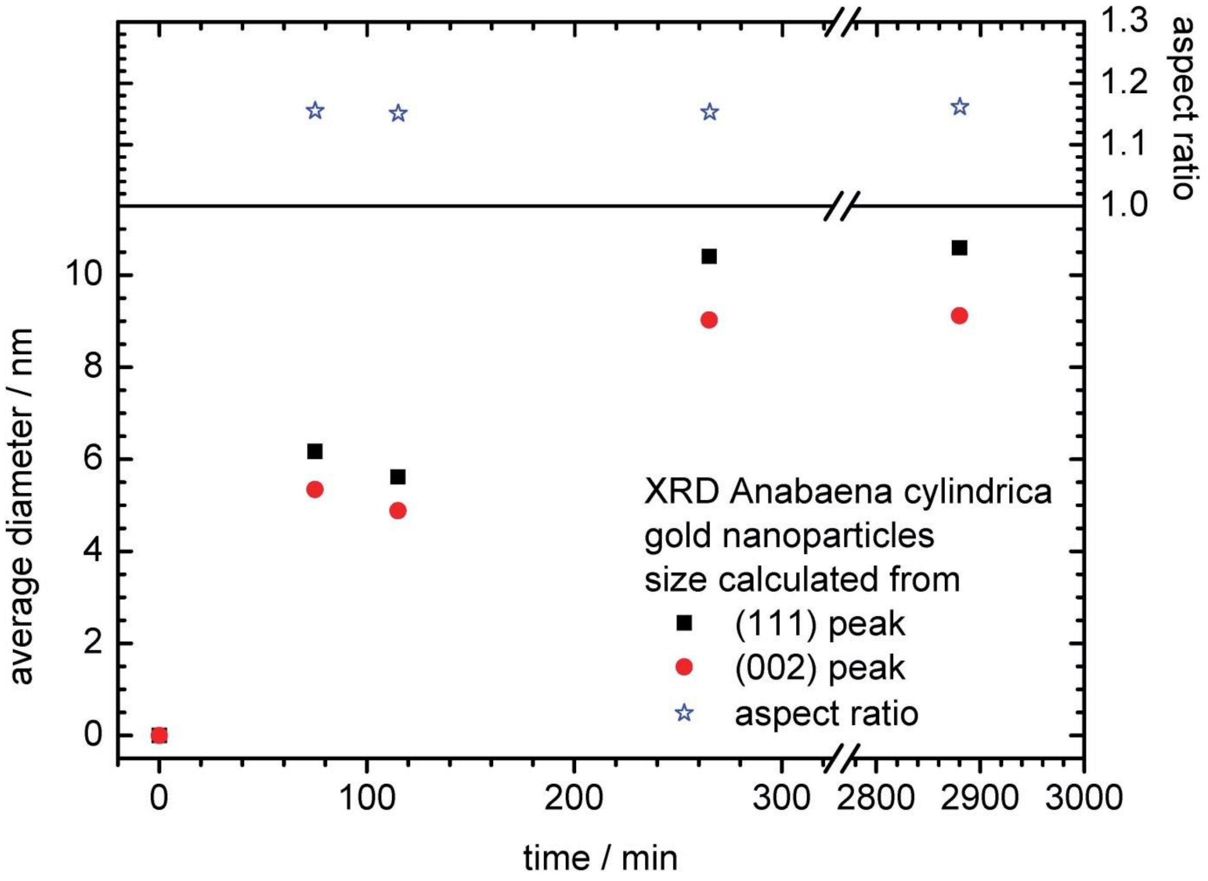

Figure S3: Average sizes of the nanoparticles produced by *Anabaena cylindrica* calculated from peak (111) in XRD (black squares) resp. (002) (red circles) over duration of incubation with 0.8 mM Au^{3+}. In the upper panel

blue stars indicate the calculated aspect ratio of an ellipsoid based on the dimensions calculated for both peaks for each individual sample.

Transmission electron microscopy

Figure S4 shows TEM of *Anabaena cylindrica*. In Figure S4a a single vegetative cell from a chain of cells is shown, in Figure S4b a heterocyst between two vegetative cells in another cell chain is visible. Thylakoid membranes (limited by light curved lines) and lipid droplets (dark dots) are clearly visible inside the cell as the EPS outside. One prominent feature of the heterocysts is the polar plugs, also called cyanophycin granules (CPG) [S5 = 28]. In the picture shown, only the polar plug at the left side of the heterocyst is visible, because of the sectional plane being not median but somewhat peripheral and slanted. Normally the CPGs are seen as an electron dense dark structure, see Figure S4b, but it is well known that it can be lost during sample preparation, leaving only an electron translucent area behind, as seen here [S5 = 28].

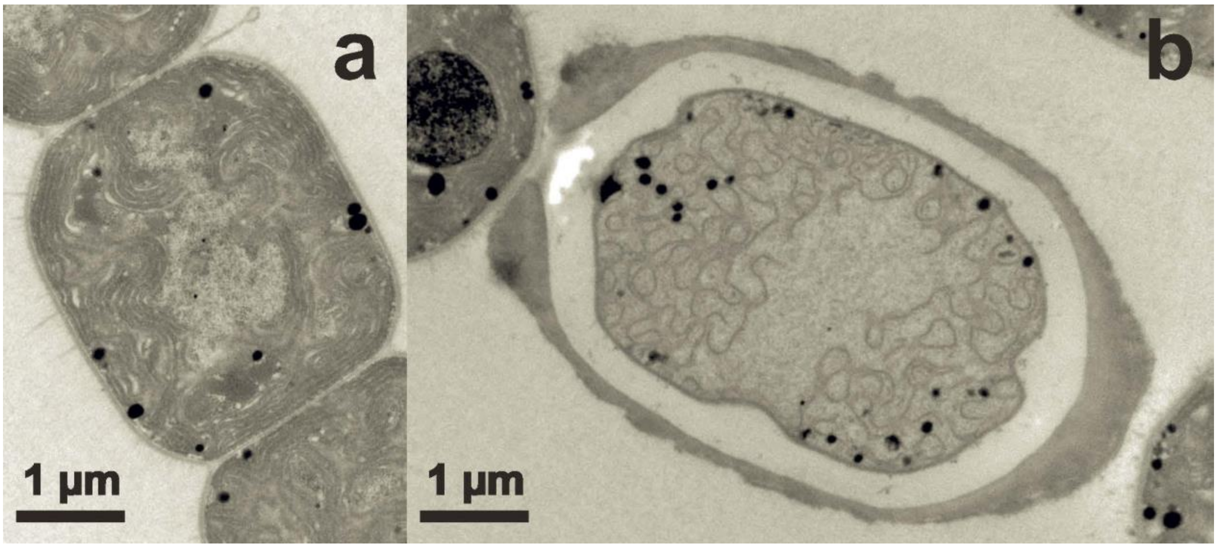

Figure S4: TEM image of a representative vegetative cell (A) and a representative heterocyst (B) of *Anabaena cylindrica*.

Since XRD detects only the average size of the formed nanoparticles, the size of each individual nanoparticle was determined from TEM images with a magnification of 50k and 85k to reveal the size distribution of the nanoparticles in specific regions of the organism using an image processing tool. The difference between the two methods is that XRD preferably detects large nanoparticles since

crystallinity is needed and image processing of TEM images is very likely to detect more very small ones because of the electron dense and structured background. One pixel in a TEM image is 1.15 nm x 1.15 nm for 85k resp. 1.94 nm x 1.94 nm for 50k. Taking this into account only nanoparticles of more than 3 nm could be detected reliably.

Laser-induced breakdown spectroscopy

A standard experimental LIBS setup as shown in **Figure S5** has been used for the experiments presented. The homemade LIBS setup consisted of a pulsed laser source (shown as a blue box in Figure S5), focusing optics (white box), and Czerny–Turner spectrometers (pink box).

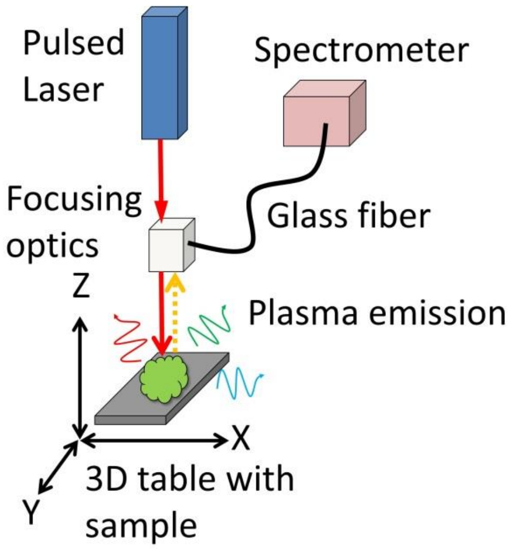

Figure S5: Schematics of a LIBS setup.

A micro-plasma (shown as green cloud in Figure S5) is generated by a low-power passively Q-switched Nd:YAG laser (CryLas, model DSS1064-3000, wavelength 1064 nm, pulse energy 2.5 mJ, pulse duration 2 ns (FWHM), repetition rate 80 pulses per second) focused on the sample surface (shown as dark gray tray in Figure S5)) by a quartz glass lens with a focal length of 20 mm.

The plasma emission (shown as yellow arrow and red, green and blue waves in Figure S5) was imaged by a combination of two quartz lenses (focal length 20 and 25 mm) into a glass fiber (core diameter 900 µm) guiding the light to a Czerny–Turner spectrometer (Ocean Optics, models MAYA 2000 Pro

(from 190 to 420 nm, spectral resolution of 0.1 nm) and USB2000 (190 to 860 nm, spectral resolution 0.3 nm)).

References

[S3] Adams, G. A.; Carr, N. G. *Crit Rev Microbiol* **1981**, 9, 45-100.

[S4] Stulp, B. K.; Stam, W. T. *Arch Hydrobiol Suppl 63/1 = Algological Studies* **1982**, 30, 35-52.

[S5 = 28] Flores, E.; Herrero, A. *Nat. Rev. Micro.* **2011**, 8, 39–50.

[S6 = 38] Zhang, C-C; Laurent, S.; Sakr, S.; Peng, L.; Bédu, S. *Mol. Microbiol*. **2006**, 59, 367–375.

[S7 = 41] Chakraborty, N.; Banerjee, A.; Lahiri, S.; Panda, A.; Gosh, N. A.; Pal, R. *J. Appl. Phycol.* **2009**, 21, 145–152.

[S8 = 60] Le Bail, A.; Duroy, H.; Fourquet, J. L. *Mater. Res. Bull.* **1988**, 23, 447–452.

[S9 = 61] Petříček, V.; Dušek, M.; Palatinus, L. *Z. Kristallogr.* **2014**, 229, 345–352.

[S10 = 62] Scherrer, P. *Nachr. Ges. Wiss. Göttingen* **1918**, 26, 98–100.

[S11 = 63] Langford, J. I.; Wilson, A. J. C. *J. Appl. Crystallogr.* **1978**, 11, 102–113.
